# Pain experience and behavior management: efficacy of photobiomodulation as an adjunct to local anesthesia in MIH patients—a randomized split-mouth clinical study

**DOI:** 10.3389/fneur.2025.1622882

**Published:** 2025-08-13

**Authors:** Aneta Olszewska, Magdalena Roszak, Aleksandra Szymczak, Elżbieta Paszyńska, Agata Czajka-Jakubowska

**Affiliations:** ^1^Department of Orthodontics and Temporomandibular Disorders, Poznan University of Medical Sciences, Poznań, Poland; ^2^Department of Computer Science and Statistics, Faculty of Medical Sciences, Poznan University of Medical Sciences, Poznań, Poland; ^3^Department of Integrated Dentistry and Community Dentistry, Poznań, Poland

**Keywords:** dental pain, pain management, molar incisor hypomineralization, photobiomodulation, local anesthesia

## Abstract

**Background:**

Pain management in children with Molar-Incisor Hypomineralization (MIH) poses challenges for pediatric dentists. MIH affects the enamel of the first permanent molars and incisors, increasing the risk of cavities, hypersensitivity, and making anesthesia and dental treatments more difficult. Children with MIH often experience dental fear, necessitating effective pain management techniques. Photobiomodulation (PBM) has potential analgesic benefits in dentistry but requires further evaluation for its effectiveness in MIH cases.

**Aim:**

This study aimed to assess the impact of photobiomodulation on pain control in maxillary permanent molars affected by MIH.

**Methods:**

In our randomized split-mouth clinical study, 45 participants (25 males, 20 females) aged 7 to 15 years, with both maxillary molars affected by MIH, were assigned to either the intervention group (PBM plus standard anesthesia, *n* = 45 teeth) or the control group (standard anesthesia alone, *n* = 45 teeth). PBM parameters included a power of 100 mW, a wavelength of 635 nm, energy of 6 J, fluence of 12 J/cm^2^, and a duration of 60 s. Pain perception, anesthesia commencement, and its extension time were measured and analyzed using SPSS version 23.0 (*p* < 0.05).

**Results:**

The PBM group reported significantly lower subjective pain (mean VAS 2 [1–5]) compared to the control group (6 [2–8]), *p* < 0.001. FLACC scale scores were also lower in the intervention group (3 [1–5]) compared to the control group (7 [5–8]), *p* < 0.001. The PBM group showed a lower heart rate (84.6 bpm ± 6.1) compared to the control (113.2 bpm ± 6.1), *p* < 0.001. Additionally, the anesthesia developed faster in the PBM group (mean: 3.6 ± 0.9 min) compared to the control (6.1 ± 0.8 min, *p* < 0.001), and the anesthetic effect persisted longer (70.2 ± 3.9 min vs. 50.7 ± 8.9 min, *p* < 0.001).

**Conclusion:**

Photobiomodulation improves pain management and the effectiveness of local anesthesia in maxillary molars affected by MIH by diminishing pain experience and enhancing anesthesia effects, providing a promising approach for pain control in children.

## Introduction

Previous dental experiences and associated pain or anxiety can negatively affect children’s cooperation during subsequent dental visits. Effective pain management ensures a positive dental attitude, encourages patient cooperation, and fosters trust in the dental environment. However, pain management methods, including local anesthetic injections, can lead to several side effects, such as discomfort, severe needle phobia, and increased general anxiety, mistrust, and nervousness in children. As a result, there has been a growing interest in alternative pain management strategies, remarkably non-invasive and minimally invasive techniques such as low-level laser therapy and acupressure, to provide painless dentistry for children (1–7).

Low-level laser therapy (LLLT), recently recommended in the literature as photobiomodulation (PBM), involves the application of low-intensity laser or diode light with specific wavelengths. It has several biological interactions with irradiated tissues, stimulating cellular activity and promoting tissue repair (8). The exact mechanism by which PBM produces its analgesic effects is not fully understood. However, it has been suggested that LLLT may provoke several mechanisms responsible for pain sensation, such as modulation of the inflammatory response in tissue, stimulation of the body’s natural pain-relieving mechanisms, and direct effects on nerve fibers (9). PBM works by inhibiting pain transmission signals through the impact on nociceptors and reducing the release of pro-inflammatory mediators like prostaglandins and cytokines. Studies have shown that PBM is more effective in reducing pain associated with C fibers than A-delta fibers, supporting the hypothesis that it may preferentially target specific nerve pathways involved in pain transmission (8, 10). This has important clinical implications. Furthermore, PBM increases the production of endogenous opioids, such as endorphins, which contribute to pain relief (11). These mechanisms make PBM a promising non-invasive alternative to conventional pain management strategies in pediatric dentistry. However, the effectiveness of LLLT may vary based on different dental procedures and individual patient characteristics related to pain and anxiety (12–17).

Molar incisor hypomineralization (MIH) is a developmental disorder that affects dental enamel, primarily impacting the newly erupted first permanent molars and permanent incisors (18). Teeth affected by MIH are more prone to caries, and defective tissues are easily fractured under normal chewing forces. Abnormal tissue morphology and composition result in hypersensitivity to external stimuli such as cold and air, and even worsen the difficulty in achieving adequate local anesthesia. This makes dental treatment challenging for this group of patients.

Data from the literature showed the prevalence of MIH from 3.6 to 25%, depending on various populations. The etiology of MIH is complex. Some etiological factors are hereditary, while others remain unexplained (19, 20). A well-balanced diet is crucial for the proper formation and mineralization of dental tissue, beginning in the embryonic period when tooth buds start to develop and continuing throughout pregnancy (21). During pregnancy and lactation, there is an increased demand not only for energy but also for a range of vital nutrients and minerals. Key minerals include calcium, phosphorus, magnesium, iron, zinc, copper, iodine, and selenium. Additionally, important vitamins that should be included are: D, A, B1, B2, niacin, choline, pantothenic acid, B6, B12, C, E, and folate (21). By managing maternal nutrition, it may be possible to mitigate the risk of dental abnormalities associated with inadequate intake of essential structural materials. Among other considerations in the study of Mafla et al., mothers’ psychological factors (depression, eating disorders, and intake of alcohol) were significantly linked to the presence of MIH (22). Other authors indicated that prenatal maternal infection, premature birth, and postnatal childhood infections (chickenpox, parotitis, measles) were significantly associated with primary teeth hypomineralization and MIH (23). There is a clear lack of direct correlation between molar incisor hypomineralization and congenital disorders. However, it is essential to recognize that during surgical procedures, mechanical trauma to the developing tooth buds may result in structural or morphological alterations. These changes could clinically resemble the hypoplasia commonly described in the literature, rather than the hypomineralization typically associated with MIH (24).

Effective management of dental procedures relies on successful local anesthesia, which is particularly important for children with MIH who may experience heightened sensitivity and discomfort even to tooth brushing (25). Hypomineralized tissues, such as altered enamel and dentin, can hinder the diffusion of anesthetics and reduce the effectiveness of local anesthesia. It clinically results in observed high pain levels and an anxious attitude in children affected by MIH. Because of these challenges, it is essential to research supplemental approaches that can enhance the pain management for this group of patients (26, 27).

Low-level laser therapy (LLLT), recently known as photobiomodulation (PBM), has emerged as a viable supplementary treatment for various medical and dental applications (28). PBM has garnered increased interest in dentistry due to its potential benefits, including enhancing the effects of local anesthesia, accelerating wound healing, and managing pain and inflammation (29). Recent research indicates that PBM increases the permeability of nerve cells, facilitating easier diffusion of anesthetic drugs, leading to faster onset and longer duration of LA. Additionally, studies have shown that PBM can alter nociceptive pathways, reducing pain perception (30–33).

Presently, the clinical studies presented the impact of Photobiomodulation Therapy (PBMT) on pain management during local anesthesia injections in children affected by MIH are scarce (32, 33). Additionally, it is emphasized that altering laser parameters, such as wavelength, energy, fluence, mode, and time of irradiation, can produce different clinical outcomes. Therefore, evaluating the analgesic effects of various laser protocols on pain control during injections is a reasonable approach (31–34).

This study evaluates the additional effect of PBM on local anesthesia efficacy in maxillary permanent molars affected by MIH.

## Materials and methods

### Study design and setting

A total of 45 children, aged between 7 and 15 years, were selected from the pediatric dentistry clinic of Poznan University of Medical Sciences in Poland for the study. The study enrolled patients with decayed maxillary molars affected by MIH on both sides, requiring invasive procedures and the administration of local anesthesia.

Ninety molars with MIH were randomly divided into two groups. In the intervention group, photobiomodulation was performed prior to the standard injection of local anesthesia, while the control group received standard anesthesia alone with the laser in off mode.

The clinical evaluation included the observation of various pain sensations and symptoms. This involved measuring heart rate (HR) and noting any accompanying symptoms during the procedure, such as sweating, skin reactions, and hyperventilation. Additionally, the patient’s self-reported pain was assessed using the test-ball squeezing test, followed by the Visual Analog Scale (VAS) and the Face, Legs, Activity, Cry, and Consolability Scale (FLACC). All findings and results were documented in the patient’s chart before and after the administration of local anesthesia.

The patient and their parents were informed about the study’s objective and the methodology to be employed. The parents or guardians of each patient who participated in this study signed a detailed and informed written consent form.

The study was conducted in the Pediatric Dentistry Clinic at Poznań University of Medical Sciences (Poland), over 24 months (2022–2024). The study followed the Declaration of Helsinki, and the local University Authority approved it (KB 235/2021, KB 429/24).

### Eligibility criteria

Children who met the following inclusion criteria were chosen to be enrolled in this study:

Inclusion Criteria:

- Patients aged 7–15 years.
- Patients with a clinically diagnosed MIH and caries affecting both maxillary permanent molars.
- Patients requiring dental treatment under local anesthesia for the affected teeth (no allergy to anesthetics/sulfites).
- Children free of any systemic disease or special health care needs.
- Children with reasonable cooperation in the dental environment (3 or 4 on the Frankel behavior rating scale).
- Completion of the written informed consent form by parents/guardians.

Exclusion Criteria:

- Patients with systemic disease/taking medication that affects dental health.
- Patients with allergies or contraindications to local anesthesia.
- Inflammation/lesion at the injection site.
- Patients with a recent history of dental treatment involving the affected molars.
- Children who have taken any analgesic drugs at least 24 h before treatment.
- Patients with dental pain (emergency).
- Uncooperative children (a previous anxious/unpleasant experience of dentistry).
- Participants who have declined to provide informed consent.

### Randomization and allocation concealment

Participants who were qualified for the treatment of maxillary MIH molars were randomly divided into one of two groups using a computer-generated list. This split-mouth study was based on the pre-anesthetic tissue management technique employed: the intervention group (receiving PBM) and the control group (receiving standard anesthesia without laser), with an equal allocation ratio of 1:1.

### Blinding

To maintain study blinding, children from both groups received the same intervention, but the control group also underwent a sham laser treatment. Additionally, the outcome assessor and the statistician remained blinded to the group assignments.

The study included the protocol of clinical evaluations, observations of physiological reactions to pain, measurements, and patient self-reports before intervention, after anesthesia, and during the treatment. Additionally, baseline data, demographic information, and general medical and dental history were recorded for each participant. The effectiveness of pain management, local anesthesia, the primary outcome measure, was assessed using the Visual Analog Scale (VAS), FLACC scale, and ball squeezing test. To obtain more objective pain measures, heart rate (HR) and physiological symptoms of pain, such as sweating, skin reactions, and hyperventilation, were recorded (35, 36). Secondary outcomes assessed in the study were the local anesthesia properties influencing pain management: LA commencement time and persistence of the anesthetic effect in the tissue.

### Clinical procedure

All clinical procedures, observations, and measurements were conducted by the same pediatric dentist to ensure consistency throughout the trial.

Patients attended two appointments within 10 days, received both injections: one with photobiomodulation and one without.

Qualification for the study included, at first, an electric pulp test (Pulp Vitality Tester, Sybron Endo, Kerr, United States) to confirm pulp vitality and exclude irreversible pulpitis. Additional periapical X-rays were performed to assess the extent of decay.

In pre-anesthesia tissue management, both groups applied at the needle penetration site a 5% Lignocaine topical anesthetic gel, Lignox (Chema Rzeszów, Poland). The anesthetic gel was left in contact with the soft tissues for 1 min to maximize its effect.

A diode laser (Smart SM Lasotronix, Piaseczno, Poland) of wavelength 635 nm was used in the intervention PBM group. The laser protocol was set with parameters as follows: power 0.1 W, energy 6 J, continuous wave, energy tip area 0.5 cm^2^, and fluence 12 J/cm^2^ was applied at the site of needle penetration for 60 s. The laser irradiation was performed in contact mode on buccal and palatal mucosa injection sites. In the control group, the laser tip was placed on the injection sites in off-mode. To ensure safety during the treatment, the patient and the operator wore safety goggles during the laser radiation and procedure. The device’s output was inspected using the aiming beam on a flat surface to maintain the parameters before each laser application.

### Local anesthesia injection

The psychological preparation for the anesthesia and procedure included a thorough explanation of each step using terminology and language suitable for a child’s age, free from any vocabulary that could imply pain.

When an understanding of the procedure and an adequate cooperation level were achieved, the child was placed supine on a dental chair, aligning their head and chest parallel to the floor, in a rest position. To avoid the child seeing the needle, the operator was holding the handpiece far from the child’s sight during LA injection. The child wore safety goggles during the whole procedure to protect from additional anxiety stimulation by light.

For the injection, Articaine hydrochloride 4% with 1:100,000 epinephrine (Citocartin 100, Molteni Dental, Italy) was chosen, using a 27-gauge disposable short dental needle (37).

The standard technique for infiltration injection was performed using the Calaject system (Ronvig, Denmark) program 2, on the tooth’s buccal gingiva, targeting the root apex’s approximate position. At the injection site, the mucosa of the cheek was stretched, and the needle was inserted between the mucobuccal fold and the mucogingival junction. A total of 1.5 mL of the anesthetic solution was deposited slowly, with a controlled speed of anesthetic, supraperiosteally. To achieve the maximum effect of local anesthesia, a palatal infiltration was administered. The palatal injection, which is typically more painful, was administered with the needle held perpendicular to the palatal surface, where approximately 0.2 mL of the anesthetic solution was deposited using program 1 of Calaject, designed specifically for this area.

The use of computer-controlled local anesthesia delivery (CCLAD) helps operators to use the same conditions (speed of anesthetic delivery, pressure on the soft tissues) during each session and for each participant, and avoid pain sensations (38–40).

### The child’s reaction to pain

The assessment of the child’s reaction to pain utilized three approaches: a physiologic method (heart rate), an objective method (FLACC scale and other pain indicators), and a subjective method (VAS scale and ball squeezing test) (41).

The heart rate (HR) was measured using the pulse oximeter placed on the patient’s index finger, and the patient was instructed to remain still and avoid moving their hands to ensure accurate readings. A baseline heart rate was recorded before local anesthesia administration, during the injection, and throughout the treatment procedures, with recordings obtained at two-minute intervals. The mean heart rate measurements were then calculated for further analysis.

The Face, Legs, Activity, Cry, and Consolability Scale was utilized to assess pain objectively. Each aspect of facial expression, leg movement, activity level, crying, and consolability was rated on a scale from 0 to 2. The total pain score was calculated as the sum of the scores from these five categories and then obtained the average during local anesthesia administration and restorative procedures (0–10). This non-verbal pain scale is typically used for young children but is also helpful for patients who cannot speak and express their pain sensations (35, 36).

The physiological reactions to pain, such as hyperventilation, sweating on the forehead, and goosebumps, were observed and documented for each patient during local anesthesia delivery and the restorative procedure.

To help manage pain sensations, patients were instructed to hold elastic balls in both hands and squeeze the right ball when experiencing moderate pain and both balls when experiencing severe pain.

**Figure 1:**
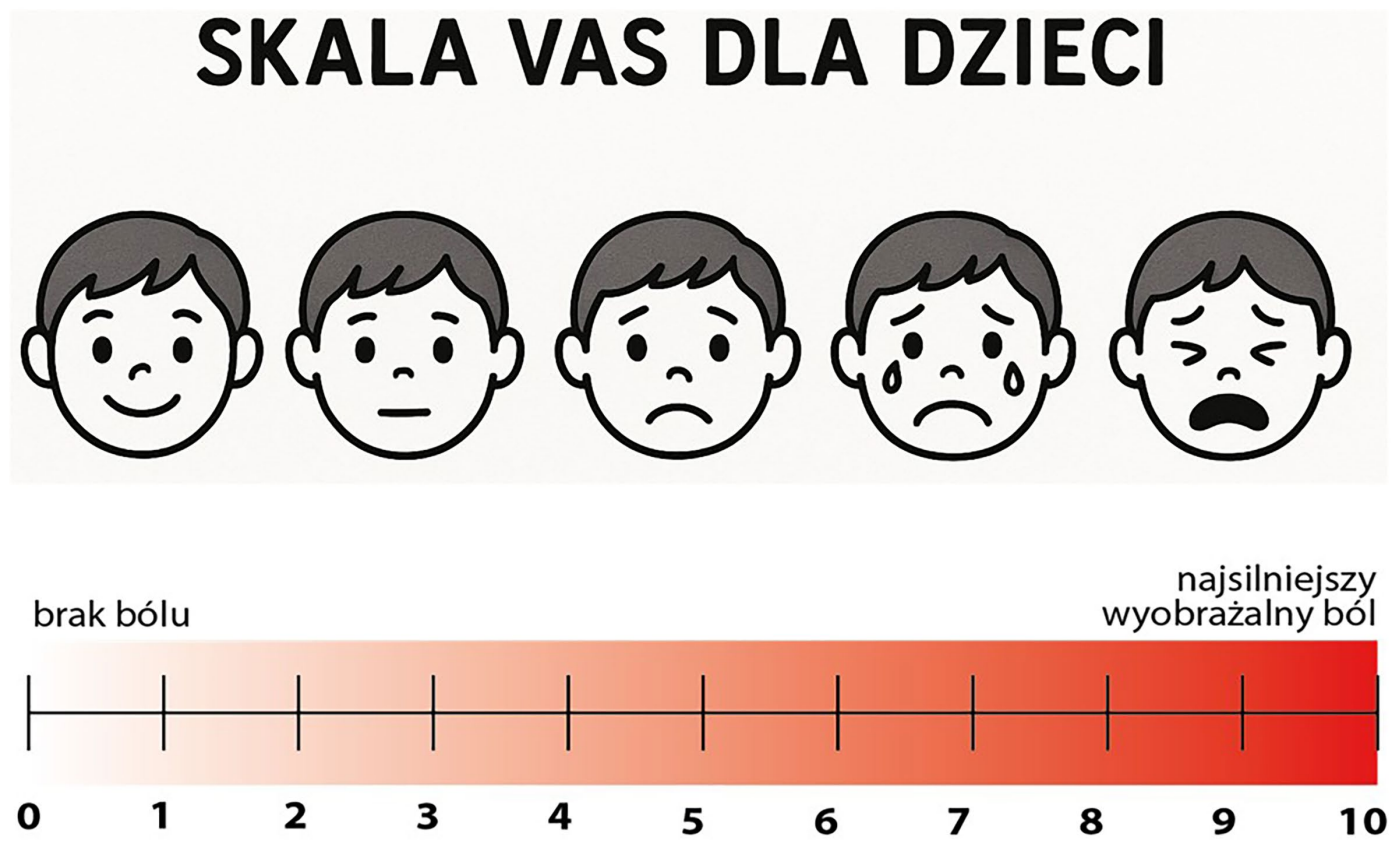
Visual Analog Scale (VAS) designed for children used in our study. Modification (use of faces) was helpful for younger children to express their pain experience. Assessment of patients’ pain experience: 0–2 = Minimal pain, 3–5 = Moderate pain, 6–10 = Severe pain.

The Visual Analog Scale form was provided to patients before any treatment, and they were explained how to use it. Instantly after the CCLAD stopped the anesthetic delivery, children were asked to estimate the pain associated with the procedure and mark it on the form from 0 to 10 (Figure 1).

### Statistical analysis

The analyzed data were expressed as mean ± standard deviation, median, minimum and maximum values, quartiles (Q1–Q3), or percentage, as appropriate. Normality of distribution was tested using the Shapiro–Wilk test. Comparison of unpaired groups was performed using the Mann–Whitney *U*-test (data did not follow normal distribution or ordinal data). Categorical data were analyzed with the *χ*^2^ test. Statistical analyses were performed with STATISTICA 13.0 (StatSoft Inc.). All results were considered significant at *p* < 0.05.

Results were presented with 95% confidence intervals, and missing data were addressed using imputation techniques to minimize bias.

**Figure 2:**
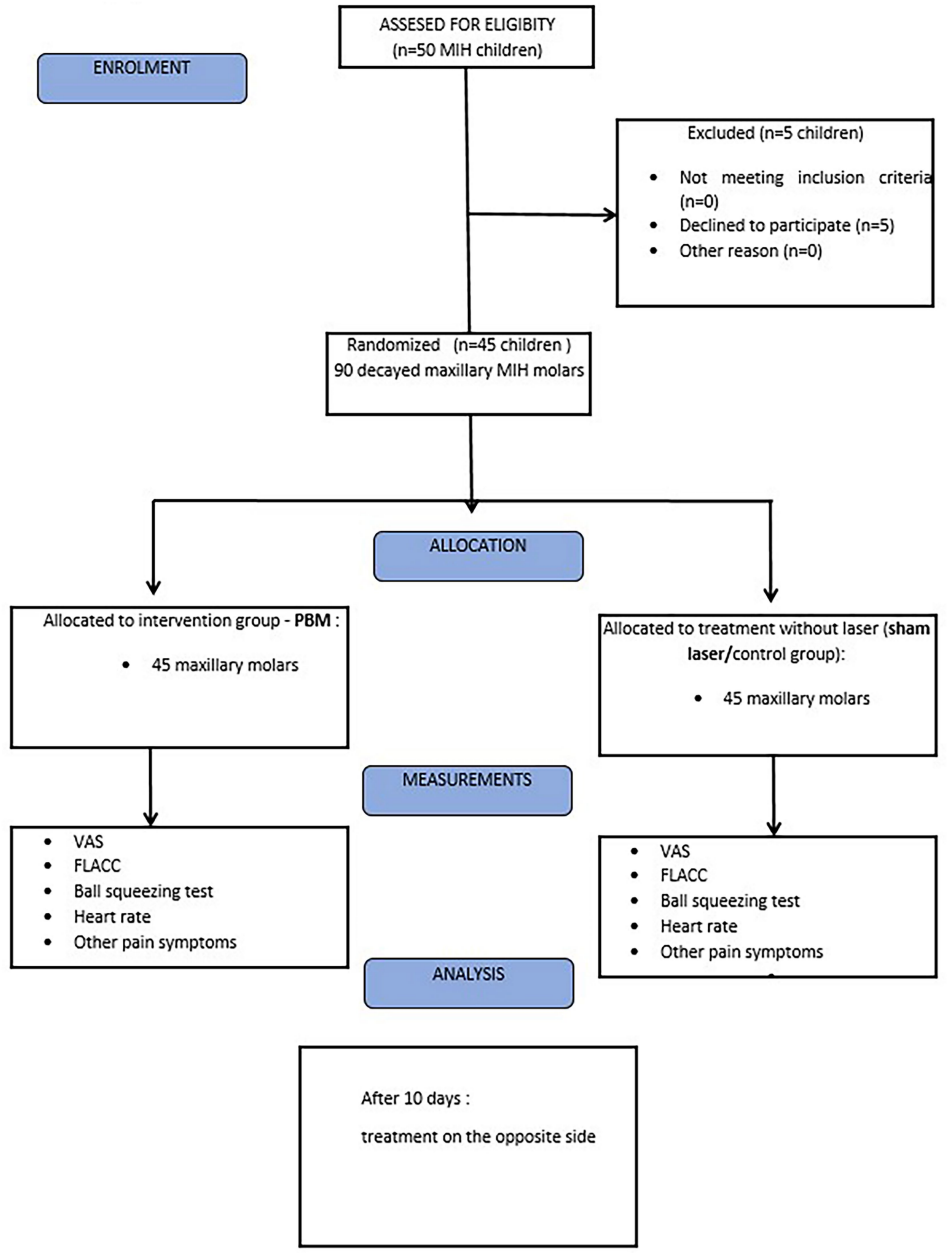
Flowchart of treated participants according to CONSORT 2010.

We have strived to transparently report the study design, methodology, and outcomes in accordance with the CONSORT guidelines where applicable (e.g., randomization, allocation concealment, outcome reporting), except prospective trial registration, as shown in the study flowchart (Figure 2).

## Results

Forty-five children diagnosed with MIH, aged between 7 and 15 years (mean age 11.2 ± 2.1) from the Pediatric dentistry clinic, Poznan, Poland, were taken for the study (Table 1). It has been reported that 60% (*n* = 27) of participants were affected by MIH, with only molars involved; hypomineralized incisors and molars were found in 40% (*n* = 18) of children.

**Table 1:** Participant demographics.

Group characteristic	Mean ± Stan. dev.
Mean age (years)	11.2 ± 2.1
Gender	*n*, %
Male	25, 55.6%
Female	20, 44.4%
Affected teeth	*n*, %
Molars	27, 60.0%
Molars and incisors	18, 40.0%

Patients with decayed maxillary molars on both the right and left sides, who were indicated for an invasive procedure requiring local anesthesia, were enrolled in the study. They were then randomized into two groups: the intervention group (which received photobiomodulation therapy in addition to standard anesthesia) and the control group (which received standard anesthesia alone). Each group consisted of 45 teeth.

The patients were scheduled for two visits within 10 days to treat both maxillary molars. The heart rate, other symptoms accompanying the procedure (sweating, skin reactions, hyperventilation), ball squeezing test, VAS, and FLACC scale were recorded in the patients before and after administration of local anesthesia.

**Figure 3:**
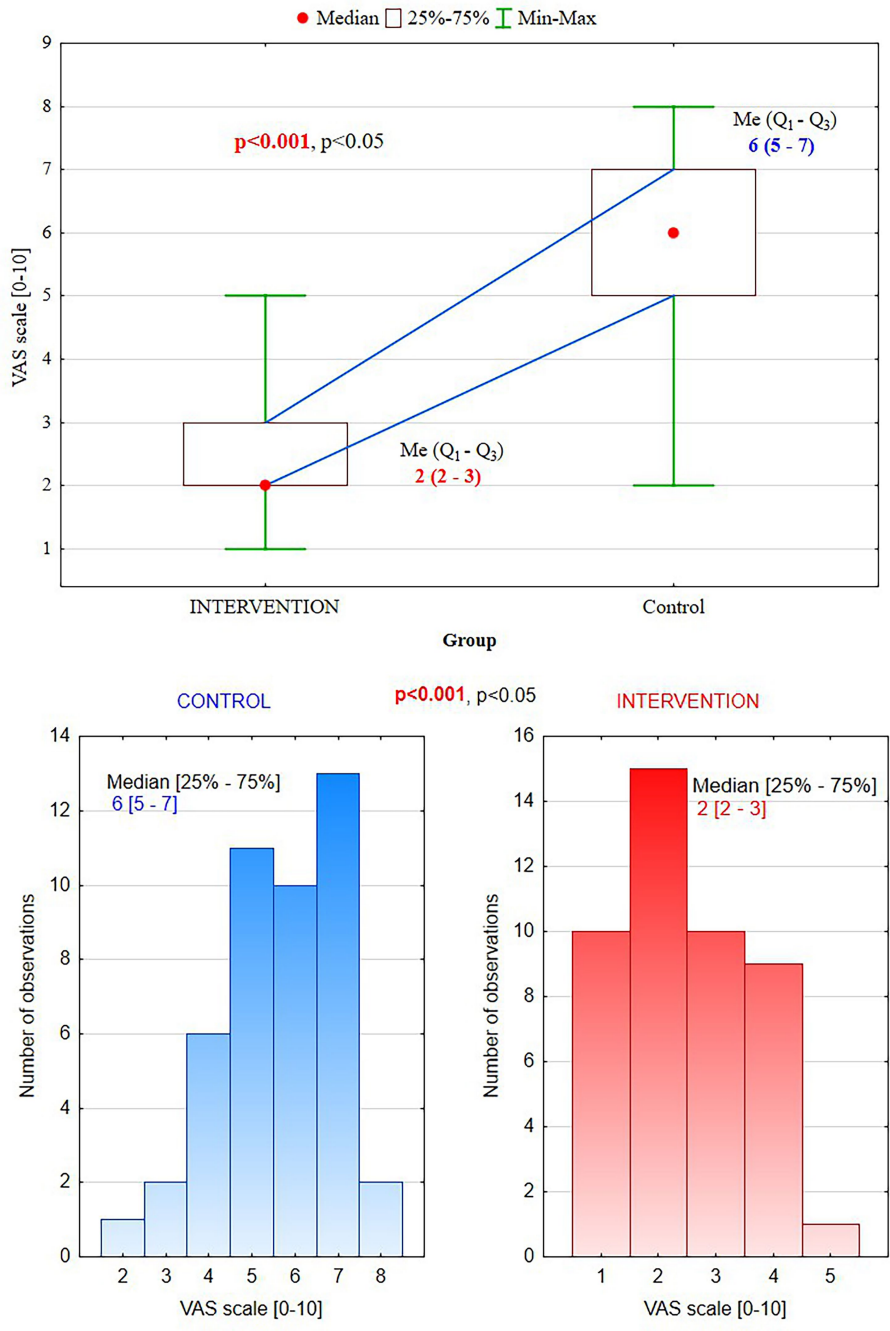
Visual Analog Scale scores: in the intervention group, 55.6% of patients reported mild pain (0–2) vs. the control group, 55.6% –severe pain (6–10).

It has been reported significantly lower pain perception in the intervention group than the control group (mean VAS score: 2.1 ± 0.8 vs. 6.2 ± 1.2, *p* < 0.001) (Table 2). Additionally, a greater proportion of participants in the intervention group reported only minimal pain (55.6%), while the control group had the same proportion reporting severe pain (55.6%) (Figure 3).

**Table 2:** Pain perception (VAS scores).

VAS score	Intervention group (n = 45)	Control group (*n* = 45)	*p*-value
Median (min–max)	2 (1–5)	6 (2–8)	<0.001
Quartiles (Q1–Q3)	2–3	5–7	
Minimal pain (VAS 0–2)	25, 55.6%	1, 2.2%	
Moderate pain (VAS 3–5)	20, 44.4%	19, 42.2%	
Severe pain (VAS 6–10)	0, 0%	25, 55.6%	

According to the FLACC scale, patients from the intervention group showed significantly lower pain perception than the control group (median values 3, vs. 7, *p* < 0.001) (Table 3).

**Table 3:** FLACC pain scale – face, legs, activity, cry, consolability–assessment of patients’ behavior (scores: 0 = Relaxed and comfortable, 1–3 = Mild discomfort, 4–6 = Moderate pain, 7–10 = Severe discomfort/pain).

FLACC value	Intervention group (*n* = 45)	Control group (*n* = 45)	*p*-value
Median (min–max)	3(1–5)	7(5–8)	<0.001
Quartiles (Q1–Q3)	2–3	6–8	

They presented only mild discomfort in the PBM group vs. severe pain in the control group, where supplemental anesthesia was needed (Figure 2).

**Figure 4:**
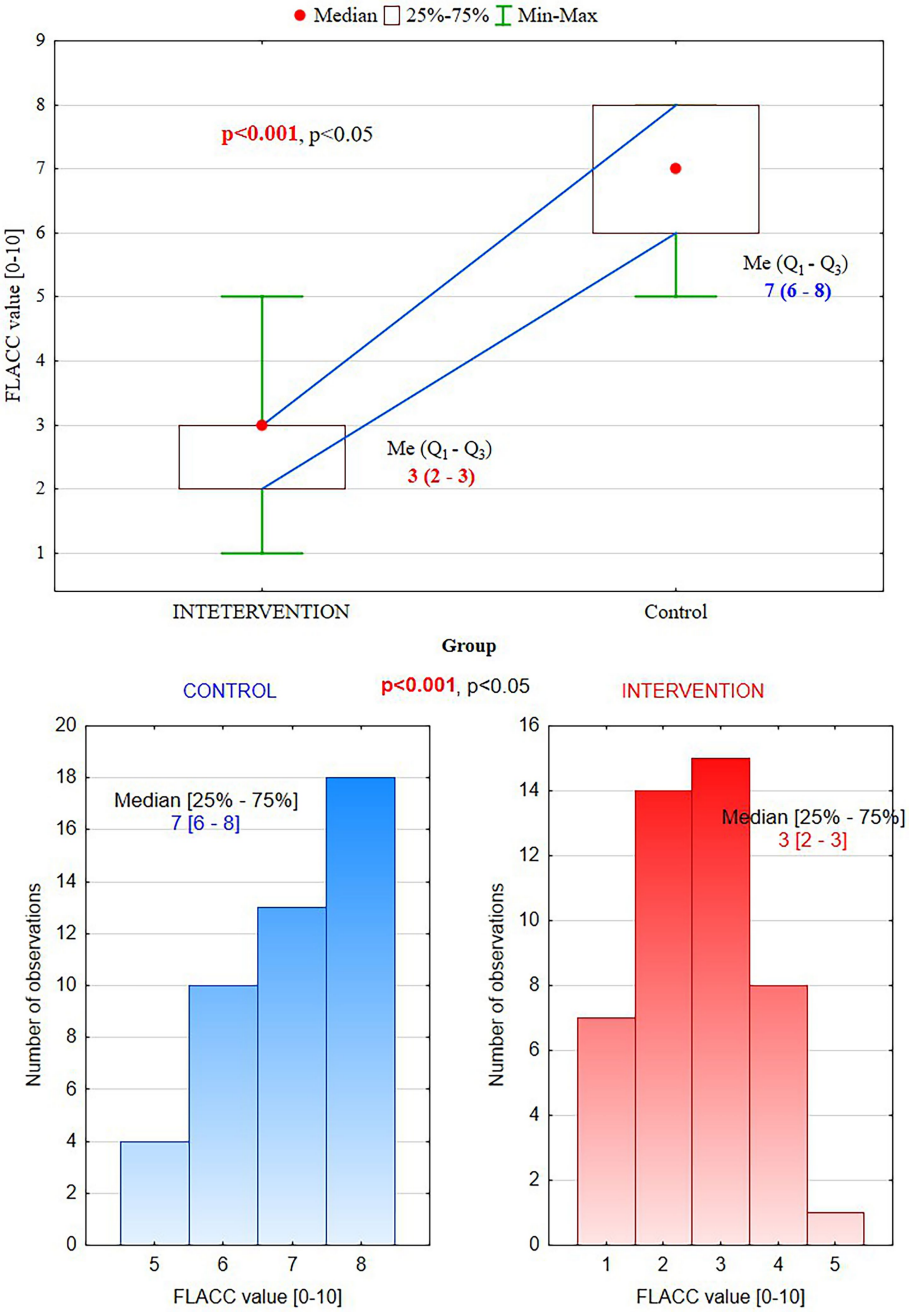
FLACC scores: in the intervention group (PBM), patients experienced mild discomfort (3) vs. severe pain (7) in the control group.

Participants in the control group represented higher mean HR scores, 113.2 ± 6.1 and 84.6 ± 6.1, respectively, in the intervention group, *p* < 0.001 (Figure 4). First measurements HR1, before PBM, showed no significant differences between groups (*p* = 0.624). However, the second measurements, H2, after PBM and local anesthesia, demonstrated significant differences, *p* < 0.001 (Table 4).

**Table 4:** Objective physiological assessment of pain sensation by heart rate (HR) measurement.

Heart rate	Intervention group (*n* = 45)	Control group (*n* = 45)	*p*-value
Mean HR score (HR1)	101.4 ± 8.9	102.4 ± 7.6	0.624
HR score range (HR1) Median (min-max)	100 (80–120)	100 (85–120)	
Mean HR score (HR2)	84.6 ± 6.1	113.2 ± 6.1	<0.001
HR score range (HR2) Median (min–max)	85 (75–100)	114 (100–120)	

HR1 – before PBM and local anesthesia, HR2–3 min after PBM and anesthesia.

Pain sensation assessed by squeezing balls held in the patient’s hands was higher in the control group, and 66.7% (*p* = 0.006) of children squeezed both balls, signaling strong pain (Table 5). In the PBM group children demonstrated little pain squeezing one ball, and only at the beginning of the clinical procedure. This test provides a practical and reliable method for assessing pain perception in pediatric patients, and the results further support the effectiveness of the intervention in reducing pain.

**Table 5:** Pain perception in ball squeezing test (little pain – squeezing ball in one hand, strong pain-squeezing ball in both hands).

Ball squeezing test (*n*, %)	Intervention group (*n* = 45)	Control group (*n* = 45)	*p*-value
1 (right side)	28, 62.2%	15, 33.3%	0.006
2 (right and left sides)	17, 37.8%	30, 66.7%	0.006

The most common symptom representing anxiety and pain was sweating observed on the forehead in 5 (11.1%) patients from the control group, followed by hyperventilation and skin reactions (Table 6).

**Table 6:** Physiological reaction to pain and anxiety.

Symptoms *n*, %	Intervention group (*n* = 45)	Control group (*n* = 45)	*p*-value
Goosebumps	1, 2.2%	4, 8.9%	0.357
Sweating	2, 4.4%	5, 11.1%	0.431
Hyperventilation	1, 2.2%	4, 8.9%	0.357

Anxiety triggers various psychophysiological responses, primarily linked to increased activity in the sympathetic branch of the autonomic nervous system. This affects several bodily systems: cardiovascular system (an increase in blood pressure and heart rate), sweat glands (heightened sweat production and increased electrical conductivity of the skin), muscles (muscle tone increases, leading to spasmodic movements and tension), respiratory system (sighing and a feeling of breathlessness), digestive system (dry mouth and constipation). All of these physiological changes can be used to assess a patient’s anxiety levels (2).

In the intervention group, a significantly faster beginning of anesthesia effect was observed than in the control group (mean onset time: 3.6 ± 0.9 min vs. 6.1 ± 0.8 min, *p* < 0.001). Additionally, the extent of anesthesia was significantly longer in the intervention group (mean duration: 70.2 ± 3.9 min vs. 50.7 ± 8.9 min, *p* < 0.001) (Table 7).

**Table 7:** Onset time and duration of anesthesia.

Parameter	Intervention group (*n* = 45)	Control group (*n* = 45)	*p*-value
Mean onset time (minutes)	3.6 ± 0.9	6.1 ± 0.8	<0.001
Median (min–max)	4(2–5)	6(4–7)	
Mean duration (minutes)	70.2 ± 3.9	50.7 ± 8.9	<0.001
Median (min–max)	71(63–81)	48(34–70)	

## Discussion

Pediatric patients in a dental office can exhibit nervousness, fear, and distrust based on their background, age, and developmental skills. In anxious situations, children may show signs of fear and agitation. These can include deep breathing, shivering, crying, or becoming withdrawn and refusing to speak or play. If the stress continues for a long time, it might affect the pain perception. Children might express their discomfort before a procedure by trying to fight or run away. This reaction can be emotionally distressing for the child, their parents, and the dental professionals involved (1–7).

Children with MIH often experience high levels of dental anxiety and difficulty tolerating treatment due to sensitivity and previous painful dental experiences. Affected enamel is softer, more porous, and prone to breakdown under regular chewing forces. Leads to post-eruptive enamel breakdown (PEB) soon after eruption when young patients are more sensitive to unpleasant situations (18, 25). Successful adhesion with recommended self-etching adhesives provides better bond strength to hypomineralized enamel than all-etch single-bottle alcohol-based adhesives. It has also been proposed to pre-treat the enamel with 5% sodium hypochlorite in order to remove intrinsic proteins encasing the hydroxyapatite and therefore facilitate etching and resin penetration. This method of covering crowns affected by MIH with composite can help reduce hypersensitivity and pain sensation in those patients (42).

Premature tooth loss necessitates orthodontic treatment, which can be particularly challenging for patients affected by MIH. Cohesive failures are common with brackets bonded to MIH-affected enamel, primarily due to reduced bond strength, which can result in enamel surface fractures when the brackets are removed. Frequent rebonding of these attachments can slow down the progress of treatment with fixed appliances (43). Considering that bonded molar tubes show a higher failure rate and more decalcification than do molar bands, it seems reasonable to choose orthodontic bands in patients with severely MIH-affected molars. It is important to emphasize that orthodontic bands can create additional areas for dental plaque retention, making proper oral hygiene more challenging and increasing the risk of caries. Severely affected teeth with MIH and extensive restorations can greatly benefit from the use of orthodontic bands, which serve as temporary retainers, maintaining these restorations for a period sufficient to postpone definitive rehabilitation after orthodontic treatment. Beyond the consideration of adhesive challenges, cases involving functional appliances using molar clasps (e.g., Twin-block) for retention may require modification of the appliance design or consideration of alternative treatment approaches (e.g., aligner technique) (44). Extracting severely affected molars at 8–10 years old has been demonstrated to result in optimal spontaneous space closure. After 10 years old, if extraction is considered, there is a higher likelihood of experiencing failure in spontaneous space closure, poor angulation, and an unsatisfactory contact point relationship with the second premolar. In addition, orthodontists should confirm the presence of the third permanent molar once studies have suggested a possible association between MIH and dental agenesis (45).

Measuring pain in children is particularly difficult. However, they often have a particular vocabulary, fewer experiences, and rely on fearsome stories from family or peers that may significantly affect their ability to express themselves, especially in a new, anxious situation. Children’s reaction to pain and ability to describe it are influenced by various factors such as their developmental stage, environment, anxiety, and psychological aspects. Therefore, it seems crucial to introduce more objective and adaptable pain measures for children (3, 38, 41, 46).

This study aimed to evaluate the potential of pre-treatment photobiomodulation to reduce the pain experienced by patients during the treatment of decayed maxillary molars affected by molar-incisor hypomineralization. PBM appears to be a promising method for minimizing pain associated with injections, providing children with a more comfortable dental experience.

Some studies have recently investigated the effect of PBM on injection pain in healthy adult patients, which is consistent with our results and explains the analgesic properties of LLLT. Presently, a few studies have addressed this topic in children, and each study represented a different protocol regarding the laser type and parameters, the method of application, mode, sample chosen, and specific teeth and anatomical area tested (46, 47). Different study protocols, methodologies, and designs made the analysis incomparable. Nevertheless, studies on MIH patients are minimal, and the results may differ according to particular hypersensitivity and pain level in this group of children (32, 33).

In our study evaluating the effectiveness of photobiomodulation, we used a diode laser with a wavelength of 635 nm and an energy density of 12 J/cm^2^ for the intervention group. It has been reported in several studies that PBM is most effective within a therapeutic wavelength range of 630–940 nm, as this range allows for deeper tissue penetration and produces beneficial biological effects in the targeted area (47). Cronshaw et al. conducted a systematic review and meta-analysis, which indicated that the recommended dose for pain relief using PBM in contact mode with tissues falls between 10 and 30 J/cm^2^ (48). According to this recommendation, we designed our laser protocol in the intervention group.

The effectiveness of laser PBM for pain relief is still under investigation. Some studies have examined the combined effect of topical anesthetic gel and laser PBM, while others have focused solely on the impact of laser PBM (31, 46, 47, 49). This diversity in research protocols has rendered the results of trials found in the literature inconsistent, highlighting the necessity and value of our study design.

We applied a 5% lignocaine gel, the most common and effective topical anesthetic in pediatric dentistry, for both the intervention and control groups. In some studies, a 20% benzocaine gel was used exclusively in the control group, while in another study, a pre-anesthetic topical gel was applied to all participants (49). Several studies have reported that the topical anesthetic benzocaine may cause allergic and toxic reactions, such as methemoglobinemia. For this reason, we chose to use lignocaine gel before injection for both group in our study (49).

Various authors have combined different techniques before administering injections to reduce pain associated with local anesthesia delivery (49, 50). Sattayut (50) conducted research to compare the effects of a 780 nm diode laser, benzocaine, local mechanical pressure, and light touch during palatal injections. They found no statistically significant differences in pain scores among the low-intensity laser (administered with an energy of 3.6 J), 20% benzocaine, mechanical pressure, and light touch (50).

The question was how the type of dental procedure might influence the analgesic effect of LLLT. Tuk et al. (51) investigated the effects of an 810 nm diode laser to assess the analgesic benefits of low-level laser therapy (LLLT) on injection sites in patients scheduled for third molar removal. They reported that the laser, with an applied energy of 5.94 J, did not significantly reduce the pain experienced during local anesthetic injections before the surgery (51).

Our study did not replicate the findings of Ghabraei et al. (52), who reported no significant difference in pain levels between the laser and placebo groups. However, our research specifically focused on patients with a history of MIH, which differs from the criteria used by Ghabraei et al. (52). In our trial, we included patients with MIH who experienced higher pain levels, and we reported differences in pain scores measured using the VAS and the FLACC scale. Also, the physiological symptoms of pain and anxiety (sweating on the forehead, hyperventilation, and goose bumps) were more common in the control group compared to the PBM group (20% vs. 8%).

Our results agreed with Shekarchi et al. (31), who revealed that laser PBM significantly decreased the injection pain. They tested the effect of PBM using an 808 nm diode laser, with 250 mW power and 32.5 J/cm^2^ fluence in contact mode for 65 s, compared to topical anesthetic gel. It has been demonstrated that the lower pain level experienced in the PBM group may be connected with the analgesic effect of the light–tissue interaction (31).

Moreover, our results did not align with those of Uçar et al. (46). Researchers were studying the effects of PBM, both alone and in combination with topical anesthesia, such as gels. In this study, an 810 nm diode laser with a power of 0.3 W and a fluence of 69 J/cm^2^ was used for 20 s in non-contact mode. They found no statistically significant difference between the two groups; however, the topical anesthesia and PBM group reported higher ‘no pain’ scores (46). In comparison to our study, they utilized different laser parameters, and their patients age and health status varied.

Furthermore, Elbay et al. (47) found no statistically significant difference in pain experiences between the group that received photobiomodulation (PBM) combined with topical anesthesia and the group that received placebo PBM and topical anesthesia. They evaluated the effects of various PBM parameters using a 940 nm diode laser in non-contact mode, with fluence levels of 69, 103, and 138 J/cm^2^ (47). The results of Elbay et al. (47) study showed no statistically significant differences in heart rate (HR), facial pain scale (FPS), and subjective evaluation of anesthesia (SEM) between the two groups during the pulpotomy and stainless steel crown (SSC) procedures. This study revealed that the effectiveness of local anesthesia was comparable in the PBM and control groups (47).

In our study, heart rate as an objective pain survey, measured before LA and during procedure, demonstrated lower mean scores after 10 min in the PBM group (84.6 bpm ± 6.1) versus the control (113.2 bpm ± 6.1). Still, there was no statistically significant difference between groups. Similarly, the ball squeezing test demonstrated higher response to pain sensation in the control group, where both balls squeezed 66.7% of children and 37.8% in the PBM group, with no statistically significant differences (*p* = 0.006).

Aykanat and Elbay (32) conducted a study on children aged 7–12 years, using photobiomodulation with a diode laser at 940 nm in continuous mode, at a power of 0.5 W and an energy density of 78 J/cm^2^. LLLT was performed before LA and planned pulp treatments (32). It has been demonstrated less pain sensation after PBM, as “no-pain” was declared by 29% of patients in the laser group and 20% in the control group. Moreover, the control group reported moderate-to-high pain levels more frequently than the experimental group (43% vs. 20%). Lower pain perception after PBM may correspond with the need for additional anesthesia. It has been noted that 49% of the control group needed supplemental injection during the treatment of teeth affected by MIH, and only 31% of the experimental group (PBM). Nevertheless, it has no statistically significant difference between the laser and control group (32).

Other studies done by Shahnawaz et al. (33) examined 70 participants with MIH. They reported higher anesthesia efficiency in the intervention group (PBM), a faster onset, and a longer duration of anesthesia with significantly lower pain perception than the control group (33). It corresponds with our findings, where in addition to more efficient pain control and lower pain perception, the intervention group reported a faster onset of anesthesia, with an average onset time of 4.2 min compared to 6.7 min in the control group. Our observations suggest that PBM combined with computer-controlled local anesthesia delivery (CCLAD) may improve the anesthesia effectiveness: faster onset and prolonged action without unpleasant sensations at the stage of initial delivery of anesthetic solution into the tissue. It has a positive impact on dental treatment in children, permitting faster initiation of dental procedures. Furthermore, the prolongation of anesthesia was higher in the intervention group, averaging 75.4 min compared to 65.3 min in the control group. The extended duration of anesthesia in the intervention group suggests that PBM may also provide better control over pain for a more extended period, which is often needed to perform a procedure in young patients.

Research indicates that PBM can effectively reduce children’s pain during dental treatments and improve patient comfort, particularly for teeth affected by MIH. It can foster a more positive view of dental care, building trust and cooperation between children and dental professionals. The study’s results showed that in the intervention group, PBM had a positive impact on pain management while treating maxillary permanent molar teeth affected by MIH.

Children who received photobiomodulation in addition to standard anesthesia reported significantly lower pain levels during dental procedures compared to those who received only standard anesthesia. The mean Visual Analog Scale (VAS) score for pain perception in the intervention group was 2.1, notably lower than the control group’s mean score of 4.5. Respectively lower pain score observed in FLACC scale in intervention group (median 3 [1–5]) and in control group median 7 [5–8], is significant, *p* < 0.001. This suggests that PBM can be an effective tool in reducing pain during dental procedures for children with MIH.

The pain perception and origin of sensations, central sensitization, and somatization are well-studied in adults; however, they have not yet been thoroughly examined in children. The reactions and perceptions of these pain phenomena in children differ markedly from those in adults. Conducting further research in this age group will provide valuable insights into effective pain management (53).

MIH strongly impacts patients at a functional and aesthetic level. Patients with this dental condition often describe not only pain and hypersensitivity, but also problems when chewing, smiling, and speaking. Esthetically, MIH has a great impact when it occurs in the anterior teeth (54). Studies showed an increase in the prevalence of caries in patients with MIH. In severe cases, MIH can lead to enamel fracture and premature tooth loss. This also has an impact on oral-health-related quality of life (55).

Based on the results of our study, PBM may be recommended as a non-pharmacological pain management technique for treating children with MIH, where local anesthesia alone is not effective. This approach of pain control minimizes pain during LA injections and fosters trust in children, ultimately leading to better behavior and cooperation during subsequent dental treatments.

## Conclusion

The results of this study indicate that photobiomodulation improves pain management in maxillary permanent molar teeth affected by molar incisor hypomineralization. Participants in the intervention group reported notably lower pain perception, as measured by the Visual Analog Scale (VAS), FLACC scale, ball squeezing test, and physiological pain symptoms. Additionally, they experienced a faster onset and longer anesthesia duration than the control group.

### Limitation

The limitations of this clinical trial include the fact that it tested only one diode laser, used a single set of laser parameters, and focused on just one anatomical site. This variability of results may be due to the varying effects of different laser parameters (wavelengths, fluence, mode, distance, time) used in each study. Future research should explore the effects of various laser parameters on the pain associated with local anesthesia injections in pediatric dental patients across different anatomical areas and types of dental procedures. Participants in our study presented molars and incisors affected by MIH, where the LA area also demands a gentle anesthesia technique. This would help validate our study’s findings and could lead to developing guidelines or protocols for managing laser photobiomodulation (PBM) in pediatric dentistry. Another limitation stems from the nature of the selected sample, as all included children were cooperative. This cooperation allowed the procedure to be completed quickly, not exceeding half the washout period for articaine. Therefore, future studies should also consider children who may be less cooperative.

## Data Availability

The original contributions presented in the study are included in the article/supplementary material, further inquiries can be directed to the corresponding author.
